# Supplementary material for: Solid matrix priming improves cauliflower and broccoli seed germination and early growth under the suboptimal temperature conditions
Source: PLoS One. 2022 Oct 3;17(10):e0275073. doi: 10.1371/journal.pone.0275073 (PMC9529278; doi:10.1371/journal.pone.0275073)
Supplement: Attachment — Submitted filename: Refree,s Report.docx [file pone.0275073.s001.docx]

Manuscript entitled: Solid matrix priming(SMP) improves cauliflower and broccoli seed germination and early growth under the sub-optimal temperature conditions

Authors: Wu Ling-Yun, Yan Jun, Huang Zheng-wu, Wan Yan-Hui, Zhu Wei-Min

Manuscript provides useful information in understanding the physiological basis of seed quality enhancement through solid matrix priming under sub-optimal temperatures in cauliflower and broccoli. Authors need to clarify the temperature range for sub-optimal conditions for these crops, since ISTA standards suggest 20 ^0^C constant temperature for conducting germination test in cauliflower and broccoli.

I think it would be more appropriate to combine results and discussion for clarity and it will also reduce duplication. The results are too brief and need to be elaborated so that the readers understand the advantages induced by solid matrix priming in improving field performance of cauliflower and broccoli seeds under sub-optimal temperatures.

See manuscript for several other comments, edits and suggestions.

The manuscript is acceptable for publication after thorough revision
