# Supplementary material for: Solid matrix priming improves cauliflower and broccoli seed germination and early growth under the suboptimal temperature conditions
Source: PLoS One. 2022 Oct 3;17(10):e0275073. doi: 10.1371/journal.pone.0275073 (PMC9529278; doi:10.1371/journal.pone.0275073)
Supplement: Attachment — Submitted filename: Manuscript PONE.docx [file pone.0275073.s002.docx]

Solid matrix priming(SMP) improves cauliflower and broccoli seed germination and early growth under the sub-optimal temperature conditions

Wu Ling-Yun*, Yan Jun, Huang Zheng-wu, Wan Yan-Hui, Zhu Wei-Min*

Horticultural Research Institute, Shanghai Academy of Agricultural Sciences, Jinqi Road, Shanghai, PR,China

*Corresponding author

E-mail: [wulingyun@saas.sh.cn(Wu](mailto:wulingyun@saas.sh.cn(Wu) Lingyun); wmzhu69@126.com(Zhu Weimin)

Competing interests: The authors have declared that no competing interests exist.

**Abstract**

Seed priming is an effective method in imparting stress tolerance to plants. This study analyzed the effects of solid matrix priming(SMP) on cauliflower and broccoli seed germination and early seedling growth under the sub-optimal temperatures. The SMP method used in this study included:(*i*) Mixing seeds with vermiculite and water at a ratio of 2:3:2.5(w/w/v) and incubated for two days in the dark at 20 °C. (*ii*)Drying the SM-primed seed. (*iii*) Germinating the SM-primed and the non-primed seeds at 10, 15, 20, and 25°C, respectively. The results showed that the solid matrix priming improved seed germination vigor and increased the activities of several antioxidant enzymes in the germinating cauliflower and broccoli seeds under the sub-optimal temperature conditions, compared with non-primed seed. Sub-optimal temperature conditions (i.e., 10, 15, and 20 °C) suppressed cauliflower and broccoli seed germination and early seedling growth. Furthermore, SMP improved seed germination capacity, and seedling growth and increased several antioxidant enzymes activities in the SM-primed germinating cauliflower and broccoli seeds under the sub-optimal temperature conditions. Inside a greenhouse, the SMP improved the emergence of cauliflower and broccoli seeds during the later winter season.SMP is an effective method for improving seed germination and emergence of cauliflower and broccoli under the sub-optimal temperature conditions.

*Additional Key words:* Seed priming; low temperature; antioxidant enzymes; *Brassica oleracea*

*Abbreviations used:* SMP, Solid matrix priming; NP, non-primed; GV, germination vigor; GR, germination rate, GI, germination index; MGT, mean germination time; MET, mean emergence time; EI: emergence Index; EDTA, ethylene diamine tetraacetic acid; SOD, Superoxide dismutase; CAT, Catalase; POD, Peroxidase; APX, Ascorbate peroxidase.

**Introduction**

Cauliflower(*B. oleracea* *L.*var.*bortytis*) and broccoli(*B. oleracea* var.*italica*) are important vegetable species and are widely cultivated and consumed. Temperature is an important factor that can affect seed germination and emergence significantly. Because cauliflower and broccoli seeds are often sown in greenhouse, seed beds, or in open field in winter or in early spring, their germination and seedling emergence are one of the major concerns of farmers. Seed priming is a method that can be used to improve seed vigor through enhancing seed germination rate and uniformity of emergence. Seed priming is, however, insufficient to promote radical protrusion[1]. Seed priming activate pre-germinative metabolic events to hasten rapid and uniform seed germination. Several studies have indicated that seed priming can induce enzyme activities and increase the production of antioxidants, biosynthesis of carbohydrates, phytohormones, late embryogenesis abundant proteins, and many other proteins, including heat-shock proteins[1,2,3,4]. In addition, priming can enhance DNA replication and repairment as well as respiratory associated pathways[5]. Seed priming has also been shown to reduce cellular damages occurred during water imbibition[1]. Consequently, seedlings derived from primed seeds often exhibited enhanced tolerance to abiotic stresses[6,7].

Seed priming is an effective method to increase plant tolerance to low temperature caused damages. For example, priming seeds with CaCl_2_ can improve wheat and maize chilling tolerance in the late-sown conditions [8]. Seed priming can significantly improve bitter gourd seed germination vigor and upregulation of multiple anti-oxidative enzymes[9]. Priming tobacco seeds with putrescine significantly increased seed germination vigor and seedling biomass when compared with non-primed control during chilling stresses[10]. Osmopriming has been shown to improve spinach germination at 5 and 20ºC, respectively[11]. Demir and Mavi reported that priming watermelon seed with KNO_3_ increased seed emergence, and improved seedling growth under the greenhouse conditions in early spring[12]. Sorghum and eggplant seeds also showed an improvement in germination attributes under low temperature conditions[13,14]. SMP combined with *Trichoderma viride* have been shown to improve okra seed emergence and fruit productivity under low temperature conditions[15].

The aim of this study was to evaluate the effects of SMP on cauliflower and broccoli seed germination and early seedling growth under the sub-optimal temperatures, and to investigate the physiological and biochemical changes in the germinating seeds.

**Materials and Methods**

**Seed material and experimental treatments**

Seeds of cauliflower cv.Husong 85 and broccoli cv. No.5 Hulv were obtained from the Shanghai Academy of Agricultural Sciences, Shanghai, China. For solid matrix priming(SMP), these seeds were mixed with vermiculite and water at a ratio of 2:3:2.5(w/w/v), and then incubated for two days in the dark at 20°C.The primed seeds were dried at 25ºC for 24h and then at 30ºC for 24h prior to further use. Seeds without SMP treatment were used as controls.

**Seed germination test**

Fifty SM-primed or non-primed control seeds were germinated at 10,15, 20 and 25°C, respectively, for seven days. This experiment was repeated three times. The germinated seeds were counted daily. At seven days after incubation, fresh weight and root length of 10 germinated seedlings were measured individually to represent each treatment. Seed germination vigor(GV), germination rate(GR), germination index(GI) and mean germination time(MGT) were calculated as per standard proceedures[16]. Each treatment had three biological replicates in this study.

**Analysis of** **antioxidant enzyme activities**

SM-primed and non-primed seeds were germinated for 17h for broccoli and 24h for cauliflower at 10, 15, 20, and 25°C, respectively, harvested, and then analyzed for antioxidant enzyme activities. The harvested SM-primed or non-primed germinating seeds were ground and extracted in ice-chilled 50mM potassium phosphate buffer, pH 7.0, with 0.1mM ethylene diamine tetraacetic acid(EDTA). The extracts were centrifuged at 17,000×*g* for 20min at 4°C, The supernatant of each sample was collected for enzyme activities determination.

Superoxide dismutase (SOD) activity was determined as described[17]. The reactions were conducted in individual tubes and illuminated for 25 min at 25ºC and absorbance was recorded at560 nm. The reactions in the tubes without illumination were used as controls. Catalase (CAT) activity was measured as described previously by Cakmak and Marschner[18]. The decrease of 240 nm absorbance for H_2_O_2_ was measured for each reaction, and used to represent the CAT activity. Peroxidase (POD) activity was determined by measuring the oxidation of guaiacol in the presence of H_2_O_2_, and the increase of 470nm absorbance over a one-minute interval. Ascorbate peroxidase(APX) activity was assayed as described[19]. The oxidation of ascorbate was induced by H_2_O_2_, and the decrease of 290 nm absorbance in every 2 min was monitored.

**Emergence test**

Emergence test using SM-primed and non-primed control seeds was performed in a glasshouse. For each treatment, two biological replicates with 50 seeds each were tested in the glasshouse. In this study, the daily minimum air temperature ranged from 3.9 to 11.8 ºC, and the daily maximum air temperature ranged from 11.4 to 34.7ºC. Seedling emergence was recorded daily for 16 days and a seed was considered as emerged when its hypocotyl appeared above the ground surface. The mean emergence time(MET) and emergence index(EI) were calculated as described below.

*EI* =∑ (*Et*/*Dt*); where *Et* is the number of emerged seeds in *t* days; *Dt* is the number of the corresponding emergence days.

*MET =∑(Et×Dt) _/_∑Et*.

**Statistical analysis**

Data were analyzed for the statistical differences using SPSS v.16. Duncan’s Multiple Range Test was used for one-way ANOVA to determine the difference among different treatments.

**Results**

**Effects on seed germination and early seedling growth**

Results showed that GV and VI of non-primed broccoli seed at 25ºC were about 85.3% and 27.1, respectively, and the MGT of the non-primed broccoli seed was about 1.8d. At 20, 15 and 10 ºC, however, the averaged GV of the non-primed broccoli seed reduced to 46.7, 0 and 0%, respectively, and the averaged VI reduced to 21.4, 13.7, and 1.1, respectively. In addition, these seeds germinated slower (Fig.1A,C,E, and G).The averaged GR of the non-primed broccoli seed reduced to 10.7% at 10ºC. In contrast, the SM-primed broccoli seed showed alleviated reductions of seed vigor at the lower temperature conditions compared with the non-primed control seed. The GRs of the SM-primed broccoli seeds were similar to the non-primed seeds at 25, 20, and 15 ºC, and maintained at 87.3% at 10 ºC. The VIs of the SM-primed broccoli seeds were significantly higher than that of the non-primed broccoli seeds, and the MGTs of the SM-primed broccoli seeds were significantly shorter than that of the non-primed seeds at all assayed temperatures. The SM-primed and non-primed cauliflower seeds also showed similar trends(Fig.1B, D, F, and H).

Fig.1 Effects of SMP on cauliflower and broccoli seed germination at 10, 15, 20 and 25°C, respectively.(A and B) Germination vigor, (C and D) Germination rate, (E and F) Vigor index, (G and H) Mean germination time. The non-primed (NP) and SM-primed (SMP) seeds were germinated on wet filter papers inside plastic boxes for seven days at 10, 15, 20 and 25 °C, respectively. Different small letters indicate significant differences between the treatments (*p*< 0.05). The same below.

In regard to root length and fresh weight, The SMP was found to be effective in promoting root length and fresh weight at 20, 15, and 10ºC compared with the non-primed seed(Fig. 2).

Fig.2 Effects of SMP on the early growth of cauliflower and broccoli seedlings at 10, 15, 20 and 25°C, respectively. (A and B) Root length, (C and D) Fresh weight. Seeds of cauliflower and broccoli were germinated as described in Figure 1.

**Effects on antioxidant enzyme activities in germinating seeds**

In this study, the SOD activity in the SM-primed germinating broccoli seeds at 25 ºC was significantly higher than that in the non-primed germinating broccoli seeds. At 20, 15 and 10 ºC, the SOD activities in the SM-primed germinating broccoli seeds were similar to that in the non-primed germinating broccoli seeds. In contrast, the SOD activity in the SM-primed germinating cauliflower seeds at 25 and 20 ºC were similar to that in the non-primed geminating cauliflower seeds. However at 10 and 15 ºC, the SOD activity in the SM-primed germinating cauliflower seeds were significantly induced compared with that in the non-primed cauliflower seeds (Fig. 3A and B). The POD activities in the SM-primed geminating broccoli seeds were consistently higher than that in the non-primed geminating broccoli seeds, especially at 10, 15 and 20ºC. In the non-primed geminating cauliflower seeds, the POD activity was always low. The POD activities in the SM-primed geminating cauliflower seeds declined from 25 to 15 ºC, and was similar to that in the non-primed geminating cauliflower seeds at 10 ºC (Fig. 3C and D). The CAT activities in the SM-primed geminating broccoli seeds were always higher than that in the non-primed geminating cauliflower and broccoli seeds except for SM-primed germinating cauliflower seed at 10℃ (Fig. 3E and F). The APX activities in the SM-primed geminating cauliflower and broccoli seeds at 15, 20 and 25 ºC were significantly higher than that in the non-primed geminating cauliflower and broccoli seeds, but was similar to that in the non-primed geminating cauliflower and broccoli seed at 10 ºC (Fig. 3G and H).

Fig.3 Effects of SMP on four antioxidant enzymes activities in the germinating cauliflower and broccoli seeds incubated at 10, 15, 20, and 25°C, respectively. (A and B) superoxide dismutase (SOD) activity, (C and D) peroxidase(POD) activity, (E and F)catalase(CAT) activity, (G and H)ascorbate peroxidase (APX) activity. In this experiment, cauliflower and broccoli seeds were germinated as described in Figure 1. At 17 hours post incubation for broccoli and 24hours post incubation for cauliflower, the non-primed (NP) and SM-primed (SMP) germinating seeds were determined for the activities of SOD, POD, CAT, and APX. For each biological replicate per treatment, a 0.5g seed sample was used for the analyses of enzymatic activities, and the experiment was repeated three times.

**Effects on seed emergence**

In early spring, the SM-primed cauliflower and broccoli seeds emerged earlier and had higher emergence percentage than that of the non-primed cauliflower and broccoli seeds (Table 1, Fig 4).For example, the emergence percentages of the non-primed cauliflower and broccoli seeds were at 45% and 21%, respectively, while the emergence percentages of the SM-primed cauliflower and broccoli seeds were at 77 and 50%, respectively (Table 1).A similar trend was observed for the emergence index between the SM-primed and non-primed cauliflower and broccoli seeds. The mean emergence time of the SM-primed of cauliflower and broccoli seeds were significantly reduced compared with the non-primed cauliflower and broccoli seeds, respectively.

Table 1 Effects of SMP on cauliflower and broccoli seed emergence

| Seeds | Treatment | Mean emergence time/d^#^ | Emergence index | Emergence percentage/% |
| --- | --- | --- | --- | --- |
| Broccoli | NP | 11.5 | 2.0 | 45.0 |
|  | SMP | 7.1﹡ | 5.8﹡ | 77.0﹡ |
| Cauliflower | NP | 13.4 | 0.8 | 21.0 |
|  | SMP | 8.3﹡ | 3.4﹡ | 50.0﹡ |

^#^,days; *, Significant at *P*<0.05.

Fig.4 Images of emergences of non-primed(NP) or solid matrix primed(SMP) cauliflower and broccoli seeds.(A)Emergence of broccoli seed.(B)Emergence of cauliflower seed. Images were taken at 16 days post sowing. Emergence test using non-primed control seeds and SM-primed seed of cauliflower and broccoli were performed in a glasshouse. For each treatment, two biological replicates with 50 seeds each were sown in a glasshouse.

**Discussion**

Seed priming is a widely adopted method to minimize the damages caused by low temperature during seed germination[9,10,12,20]. In the present study, we have determined that cauliflower and broccoli seed germination and emergence performance under the sub-optimal temperatures can be significantly improved using the solid matrix priming (SMP). Antioxidant enzymes play important roles in improving plant chilling tolerance[21]. Antioxidant enzymes, including SOD, POD, CAT and APX, have been shown to protect cell membranes against damages caused by reactive oxygen species[10,22]. In this study, we have determined that after the SMP, the activities of POD, CAT, and APX were generally increased under the sub-optimal temperature conditions (i.e., 10, 15, and 20ºC), except the CAT activity in the SM-primed cauliflower seeds at 10 ºC, and the APX activity in the SM-primed cauliflower and broccoli seeds at 10 ºC.

In addition to seed germination and the enzymatic activity assays in the laboratory, we also conducted assays inside a glasshouse in early spring. The result showed that the SM-primed cauliflower and broccoli seeds emerged earlier and at a higher emergence percentage compared with the non-primed cauliflower and broccoli control seeds. This finding agreed with previous reports, and further confirmed that the SMP treatment can improve seed germination performance and increase the activities of multiple antioxidant enzymes. For example, the antioxidation activity of primed bitter gourd seeds germinated under the sub-optimal temperatures was increased (Wang et al., 2003). SMP treatment also improved the germination of okra seeds under a sub-optimal temperature(15ºC) inside the laboratory, and reduced days needed to germinate from 12 to 8.5 days(Pandita et al., 2010). Hydropriming, osmopriming, and SMP had also been reported to enhance the activities of multiple antioxidant enzymes in okra seeds[23]. Consequently, we conclude that the SMP can improve the overall performance of seeds, including faster and more uniformed seed germination and better emergence percentage under the sub-optimal temperatures(10 to 20 ºC). These improvements are likely due to the increased activities of antioxidant enzymes. The increased antioxidant enzymatic activities minimize the damages caused by oxidative stresses. Further analyses of physiological and molecular pathway changes in the SM-primed and non-primed seeds can help to reveal the mechanism controlling seed germination as well as early seedling growth.

**Conclusion**

In summary, our results have shown that under the sub-optimal temperature conditions(i.e.,10, 15, and 20ºC),cauliflower and broccoli seed germination vigor, germination index, seedling fresh weight and root length were reduced, especially the seed germination rate at 10 ºC. Our results have also shown that the SMP treatment can increase GI and VI, while decrease MGT when compared with that shown by the non-primed control seeds. In addition, SMP increases the activities of CAT, POD, and APX in the SM-primed germinating cauliflower and broccoli seeds. The SM-primed cauliflower and broccoli seeds also showed better emergence percentage under the sub-optimal temperature conditions in early spring. Therefore, we recommend SMP is a useful method to improve seed germination and emergence of cauliflower and broccoli under the sub-optimal temperature conditions.

**Acknowledgements**

The research was supported by Shanghai green leafy vegetables industry technology system and the Shanghai Agriculture Applied Technology Development Program, China(Grant No.G2016060105). The authors would like to thank Dr. X.S.Ding for his help on the language of this manuscript.

**Author Contributions**

Both authors designed, drafted and wrote the paper.

**References**

1. Rakshit A, Singh HB. Adances in seed priming || Stimulating plant tolerance against abiotic stress through seed priming. Springer. 2018.
2. Chen K, Arora R. Priming memory invokes seed stress-tolerance. Environmental Experimental Botany. 2013; 94:33-45.
3. Varier A, Vari AK, Dadlani M. The subcellular basis of seed priming. Current science. 2010; 99:450-456.
4. Wojtyla Ł, Lechowska K, Kubala S, Garnczarska M. Molecular processes induced in primed seeds-increasing the potential to stabilize crop yields under drought conditions. Journal of Plant Physiology. 2016; 203:116-126.
5. Thornton JM,Collins ARS, Powell AA. The effect of aerated hydration on DNA synthesis in embryos of *Brassica oleracea* L. Seed Science Research. 1993; 3:195-199.
6. Jisha KC, Vijayakumari K, Puthur JT. Seed priming for abiotic stress tolerance: an overview. Acta Physiologiae Plantarum. 2013; 35:1381-1396.
7. Sen A, Puthur JT, Seed priming-induced physiochemical and molecular events in plants coupled to abiotic stress tolerance: An overview, in:Hossain, M.A., Liu F., Burritt,D., Fujita M., Priming-Mediated Stress and Cross-Stress Tolerance in Crop Plants. Elsevier Inc., Amsterdam, 2020;pp. 303-316.
8. Farooq M, Aziz T, Basra SMA, Wahid A, Cheema MA. Exploring the Role of Calcium to Improve Chilling Tolerance in Hybrid Maize. Journal of Agronomy＆Crop Science. 2010; 194:350-359.
9. Wang HY, Chen CL, Sung JM. Both warm water soaking and matriconditioning treatments enhance anti-oxidation of bitter gourd seeds germinated at sub-optimal temperature. Seed Science ＆ Technology 2003; 31:47-56.
10. Xu S, Hu J, Li Y, Ma W, Zheng Y, Zhu S. Chilling tolerance in *Nicotiana tabacum* induced by seed priming with putrescine. Plant Growth Regulation. 2011; 63:279-290.
11. Chen K, Arora R, Arora U. Osmopriming of spinach (*Spinacia oleracea L. cv. Bloomsdale*) seeds and germination performance under temperature and water stress. Seed Science Technology. 2010; 38:45-57.
12. Demir I, Mavi K. The effect of priming on seedling emergence of differentially matured watermelon (*Citrullus lanatus* (Thunb.) Matsum and Nakai) seeds. Scientia Horticulturae 2004; 102:467-473.
13. Tiryaki I, Buyukcingil Y. Seed priming combined with plant hormones: influence on germination and seedling emergence of sorghum at low temperature. Seed Science ＆ Technology 2009; 37:303-315.
14. Zhang Y, Liu H, Shen S, Zhang X. Improvement of eggplant seed germination and seedling emergence at low temperature by seed priming with incorporation SA into KNO_3_ solution. Frontiers of Agriculture in China. 2011; 5:534-537.
15. Pandita VK, Anand A, Nagarajan S, Seth R, Sinha, SN. Solid matrix priming improves seed emergence and crop performance in okra. Seed Science＆Technology. 2010; 38:665-674.
16. Wu L, Huo W, Yao D, Li M. Effects of solid matrix priming (SMP) and salt stress on broccoli and cauliflower seed germination and early seedling growth. Scientia Horticulturae. 2019; 255:161-168.
17. Giannopolitis CN, Ries SK. Superoxide dismutases: I. Occurrence in higher plants. Plant Physiology. 1977; 59:309-314.
18. Cakmak I, Marschner H. Magnesium deficiency and high light intensity enhance activities of superoxide dismutase, ascorbate peroxidase, and glutathione reductase in bean leaves. Plant Physiology. 1992; 98:1222-1227.
19. Nakano Y, Asada K. Hydrogen Peroxide is Scavenged by Ascorbate-specific Peroxidase in Spinach Chloroplasts. Plant cell physiology. 1981; 22:867-880.
20. Sheteiwy M, Shen H, Xu J, Guan Y, Song W, Hu J. Seed polyamines metabolism induced by seed priming with spermidine and 5-aminolevulinic acid for chilling tolerance improvement in rice (Oryza sativa L.) seedlings. Environmental Experimental Botany. 2017; 137:58-72.
21. Blokhina O, Virolainen, E, Fagerstedt, KV. Antioxidants, Oxidative Damage and Oxygen Deprivation Stress: a Review. Annals of Botany. 2003; 91:179-194.
22. Bowler C, Montagu MV, Inze D. Superoxide Dismutase and Stress Tolerance. Annual Review of Plant Physiology and Plant Molecular Biology. 1992; 43:83-116.
23. Sharma AD, Rathore SVS, Srinivasan K, Tyagi RK. Comparison of various seed priming methods for seed germination, seedling vigour and fruit yield in okra (*Abelmoschus esculentus* L. Moench). Scientia Horticulturae. 2014; 165:75–81.
