# Supplementary material for: Solid matrix priming improves cauliflower and broccoli seed germination and early growth under the suboptimal temperature conditions
Source: PLoS One. 2022 Oct 3;17(10):e0275073. doi: 10.1371/journal.pone.0275073 (PMC9529278; doi:10.1371/journal.pone.0275073)
Supplement: Attachment — Submitted filename: Response to Reviewers.docx [file pone.0275073.s004.docx]

Response to Reviewer #1,

Thanks for your comments on our paper. We have revised our paper according to your comments.

10, 15 ^0^C is sub-optimal conditions for conducting germination test in cauliflower and broccoli.

We have combined the results and discussion and revised the results part.

1. *10 ^o^C and15 ^o^C is a sub-0ptimal temperature. 20 ^o^C should be optimal temperature for cauliflower and broccoli seed germination. Needs clarification*

Yes, 10 ^o^C and15 ^o^C is a sub-0ptimal temperature. Please see the revised manuscript.

2、*Should mention the benefit obtained by SMP under suboptimal temperatures over non primed seed*

I have revised. Please see the revised manuscript.

3*、Needs elaboration and clarity.*

I have revised. Please see the revised manuscript.

4、*Improvement with which method of priming ?*

I have revised. Please see the revised manuscript.

*5、What was the initial moisture content of seed prior to priming ?*

I have revised. Please see the revised manuscript.

*6、Follow ISTA protocols for germination test. Specify make of incubators/ germinator used for conducting germination test.*

Broccoli and cauliflower seed used in my experiment germinated fast. After germinate for 7 days, there were no new seed germinated.

I have revised others. Please see the revised manuscript.

7*、Too brief: the results between primed and non-primed seeds may be compared based on percent increase or decrease.*

I have revised others. Please see the revised manuscript.

*8、Significantly what? explain*

Because change of SOD activity is somewhat complexed in our study, I have deleted this sentence. Please see the revised manuscript.

*9、Describe the early spring conditions in terms of min./ max. temperatures. Whether emergence test was conducted outside in the field during early spring. If not then we may say under greenhouse conditions.*

I have revised others. Please see the revised manuscript.

*10、Mention sub-optimal temperatures.*

I have revised. Please see the revised manuscript.

Response to Dr.Mannan,

Thanks for your comments on our paper. We have revised our paper according to your comments.

1. *Mention the design of experiment with number of replication:*

I have revised. Please see the revised manuscript. About number of replication, seed germination test, analysis of antioxidant enzyeme and emergence test have different replications, please see them in materials and methods part.

*2. mention the design of experiment:*

I have revised. Please see the revised manuscript.

*3. Detailed results are not presented:*

I have revised. Please see the revised manuscript.

*4.Discussion is very poor:*

I have revised. Please see the revised manuscript.

*5.Decribe how anti-oxidant activities increased due pring using vermiculite?*

I have revised. Please see the revised manuscript.

5.what are the mechanisms?

I have revised. Please see the revised manuscript.

*6.More citation need*

I have revised. Please see the revised manuscript.
