# Supplementary material for: Solid matrix priming improves cauliflower and broccoli seed germination and early growth under the suboptimal temperature conditions
Source: PLoS One. 2022 Oct 3;17(10):e0275073. doi: 10.1371/journal.pone.0275073 (PMC9529278; doi:10.1371/journal.pone.0275073)
Supplement: Attachment — Submitted filename: Response to Reviewers.docx [file pone.0275073.s005.docx]

Response to Reviewer ,

Thanks for your comments on our paper. We have revised our paper according to your comments.

**Effects on seed emergence part:**

1、Seed priming is a widely adopted method to minimize the damages caused by low temperature during seed germination[10-11,13,21-23].

Because I increased reference 12. ***So I change it to:***

**Seed priming is a widely adopted method to minimize the damages caused by low temperature during seed germination[10-13,21-23].**

**References part:**

1、 reference 2: I deleted “,” and increased “space”.
2、 Reference 3: I increased “Singapore.”

3、reference7: I increased “space”.

4、reference8: I changed it to : Hossain MA, Liu F, Burritt D, Fujita M. Priming-Mediated Stress and Cross-Stress Tolerance in Crop Plants. Elsevier Inc. Amsterdam. 2020.

5、reference10: I deleted “space” “space”and increased “.”

6、reference12: I increased “＆”,and deleted “space”.

7、reference13: I increased “.”

8、reference15: I deleted “space”， “space” and increased “.”

9、reference24: I deleted “,”and“,”

10、reference26: I deleted “,” “,”

11、reference29: I deleted “,”

12、reference30: I deleted “.” “.” “.” “.”.

13、reference31: I deleted “.” “.” “.”.

Please see the revised manuscript.
