# Supplementary material for: Solid matrix priming improves cauliflower and broccoli seed germination and early growth under the suboptimal temperature conditions
Source: PLoS One. 2022 Oct 3;17(10):e0275073. doi: 10.1371/journal.pone.0275073 (PMC9529278; doi:10.1371/journal.pone.0275073)
Supplement: Attachment — Submitted filename: Response to Reviewer and Editor.docx [file pone.0275073.s006.docx]

Response to Editor,

Thanks for your comments on our paper. We have checked and revised the grammar of the manuscript according to your comments.

Line 1: I increased “space” between priming and (SMP)

Line 3: I deleted “space”

Line 7: I increased “space” between E-mail: and [wulingyun@saas.sh.cn](mailto:wulingyun@saas.sh.cn%20(Wu)

Line 12: I increased “space” between priming: and (SMP)

Line 14: I increased “space”

Line 15: I deleted “space” and increase “space”

Line 24: I deleted “space”

Line 25: I deleted “cauliflower and broccoli”

Line 26: I increased “of cauliflower and broccoli”

Line 39: I increase“5 space”

Line 40: I deleted “are and space”.

Line 45: I increased “space” between emergence and [1-2].

Line 48: I increased “space” between protrusion and [3].

Line 52: I increased “space” between proteins and [3-6].

Line 53: I increased “space” between pathways and [7].

Line 54: I increased “space” between imbibition and [3].

Line 56: I increased “space” between stresses and [8] and deleted “space”.

Line 57: I changed “increase” to “improve”.

Line 58: I deleted “caused damages”.

Line 59: I increased “space” between conditions and [9].

Line 61: I increased “space” between enzymes and [10].

Line 64: I increased “space” between respectively and [12].

Line 66: I increased “space” between spring and [13].

Line 67: I increased “space” between growth and [14].

Line 69: I increased “space” between conditions and [15].

Line 71: I increased “space” between conditions and [16].

Line 80, 81 and 82: I increased “space” respectively.

Line 85: I changed “flilter” to “filter”.

Line 86: I changed “were” to “was”.

Line 87: I increased “space”.

Line 90: I changed “seven” to “seventh”.

Line 92 and 93: I increased “4 space”.

Line 95: I increased “2 space”.

Line 98: I increased “2 space”.

Line 99: I increased “space”.

Line 100: I deleted “，” and increased “,”.

Line 102: I increased “space”.

Line 105: I increased “space”.

Line 107, 108 and 109,: I increased “space” respectively.

Line 116: I deleted “space”.

Line 119: I increased “2 space”.

Line 121: I changed “in” to “at”.

Line 132: I deleted “space”.

Line 134: I increased “space”,deleted “.”and increased “2 space”.

Line 138: I deleted “2 space”.

Line 146: I deleted “space”, increased “space”, and increased “, respectively”.I deleted “significantly increased from 0 to”, deleted “space”, increased“, while”, deleted “compared with”, and increased “did not germinate”, deleted “.”, increased “space”.

Line 150: I changed “Fig.1” to “Fig 1.”

Line 151: I increased “space”

Line 153: I deleted “space”

Line 157 and 158: I deleted “space”

Line 160: I deleted “space”and “.”

Line 162: I changed “Fig.2” to “Fig 2.”

Line 166: I changed “activity” to “activities”

Line 167: I changed “was” to “were”

Line 168: I deleted“space”

Line 169: I changed “activity” to “activities”

Line 170: I deleted“space”

Line 171~189: I changed“geminating” to “germinating”, changed “was” to “were”, changed“Fig.3” to “Fig 3.”, changed “seed” to “seeds”, increased “space” and deleted “.”

Line 190~192: I increased“Each treatment had three biological replicates in this study.”, deleted“ For each biological replicate per treatment, a 0.5g seed sample was used for the analyses of enzymatic activities, and the experiment was repeated three times.”

Line 194~214: I deleted “in a glasshouse”, “space”, “of”, increased “space”, and changed “Fig.4” to “Fig 4.”

Line 215~249: I increased “activities of”, changed “activity” to “enzymes”, changed “was” to “were”, changed “There was an early reduction of activity of unprimed and SM primed spinach seeds were repressed during germination(0-5) , followed by a significant increase in CAT by 10 d germination” to “There was an early reduction of activity in CAT activity during germination(0-5 d), followed by a significant increase by 10 d of germination.”, increased “space” and deleted“space”.

Line 250~264: I deleted “a”, “in early spring”, and increased “space” and deleted “space”.

Line 277~280: I increased “space”, and changed “Both” to “All”.

Please see the revised manuscript.

Yours sincerely,

Lingyun Wu

E-mail:wulingyun@saas.sh.cn
