# Supplementary material for: Solid matrix priming improves cauliflower and broccoli seed germination and early growth under the suboptimal temperature conditions
Source: PLoS One. 2022 Oct 3;17(10):e0275073. doi: 10.1371/journal.pone.0275073 (PMC9529278; doi:10.1371/journal.pone.0275073)
Supplement: Attachment — Submitted filename: Response to Reviewers.docx [file pone.0275073.s007.docx]

Response to Reviewers,

Thanks for your comments on our paper. We have contacted English editing sevice company to check the language according to your comments.

Please see the revised manuscript.

Yours sincerely,

Lingyun Wu

E-mail:wulingyun@saas.sh.cn
